# Revisiting What Constitutes a Neglected Tropical Disease?

**DOI:** 10.1371/journal.pntd.0012794

**Published:** 2025-02-20

**Authors:** Paul J. Brindley, Peter J. Hotez, Shaden Kamhawi

**Affiliations:** 1 Department of Microbiology, Immunology and Tropical Medicine, School of Medicine & Health Sciences, George Washington University, Washington, DC, United States of America; 2 Departments of Pediatrics and Molecular Virology & Microbiology, Texas Children’s Hospital Center for Vaccine Development, National School of Tropical Medicine, Baylor College of Medicine, Houston, Texas, United States of America; 3 Hagler Institute for Advanced Study, Texas A&M University, College Station, Texas, United States of America; 4 Department of Biology, Baylor University, Waco, Texas, United States of America; 5 James A Baker III Institute of Public Policy, Rice University, Houston, Texas, United States of America; 6 Scowcroft Institute of International Affairs, Bush School of Government and Public Service, Texas A&M University, College Station, Texas, United States of America; 7 Vector Molecular Biology Section, Laboratory of Malaria and Vector Research, National Institute of Allergy and Infectious Diseases, National Institutes of Health, Bethesda, Maryland, United States of America

## Abstract

*PLOS Neglected Tropical Diseases* (PLOS NTDs) publishes research devoted to pathogenesis and other clinical aspects, epidemiology, prevention, diagnosis, treatment, and control of the neglected tropical diseases (NTDs), as well as work relevant to public health policy. We define NTDs as poverty-promoting infectious diseases that can negatively impact the quality of life in rural areas and poor urban areas of low- and middle-income countries but which can also affect specific communities within high-income countries. The poverty-inducing effects of the NTDs operate by impairing child health and development, pregnancy outcomes for both mother and child, worker productivity, and quality of life. The NTDs also exhibit stigmatizing features, impeditive to equity and economic stability. The objective of this editorial is to provide an update on the conditions that PLOS NTDs will consider for publication.

## Revisiting our Scope

Starting with the first issue of PLOS NTDs in 2007, our community of public health and scientific experts has worked to refine and shape the meaning of “neglected tropical diseases (NTDs)”, emphasizing both their occurrence in the setting of poverty and their role in reinforcing poverty through their often chronic, debilitating, and stigmatizing features [1]. From the first global partners meeting in 2007 and to date, our list of NTDs incorporated those conditions defined by the World Health Organization (WHO) and their Department of NTDs in https://www.who.int/health-topics/neglected-tropical-diseases#tab=tab_1. However, we noted the urgency of including additional diseases based on the needs and wishes of the NTDs community.

Five years ago, we wrote an editorial entitled “What constitutes a Neglected Tropical Disease” [2] with the intention of explaining to the scientific community and wider public our ambition for this journal. Recognizing the evolving landscape of NTDs, we decided to periodically revisit the scope of the journal to ensure that we stay current and up to date in service of our community. Each time, this exercise has two overriding considerations: 1) The need to require some key distinguishing features of these conditions to consolidate and maintain the identity of the NTD community while enhancing inclusion, and 2) avoid narrowing the definition to the point where those afflicted by important poverty-promoting conditions are inadvertently ignored or neglected. That aside, rare diseases that do not represent a public health problem will not be considered.

During these five years, several newsworthy advances have taken place notwithstanding the interruption from the COVID-19 pandemic. In response to our invitation to identify neglected, emerging, or expanding conditions for potential publication in PLOS NTDs, several editorial board members and external experts have contributed viewpoints and editorials. These have highlighted overlooked contributors to morbidity, mortality, and economic loss, including the impact of social stigma on neglected and marginalized populations, recommending additional pathogens and diseases for inclusion.

PLOS NTDs current disease categories include: i) Helminth diseases; ii) Protozoal diseases; iii) Viral diseases, especially those caused by arboviruses; iv) Bacterial diseases; v) Vector biology and control; vi) Fungal diseases; vii) Non-infectious diseases; and viii) One Health and Health Policy. It is important to note that whereas almost all helminth and protozoal illnesses are considered an NTD, only selected viral, bacterial, and fungal infections fall in-scope based on their unique high endemicity in disadvantaged tropical regions or low-income countries. Under these general categories, additional diseases now in scope at PLOS NTDs include envenoming beyond snakebite [3], Noma [4, 5], Rickettsial infections [6] including Rocky Mountain spotted fever [7], SARS-CoV-2 [8], mpox [9, 10], Dabie bandavirus (severe fever with thrombocytopenia syndrome [SFTS] virus) [11, 12], West Nile Virus [13], Strep A *Streptococcus* [14] and an expansive and expanding catalog of fungal diseases that includes talaromycosis [15], systemic mycoses [16], and increasingly drug-resistant dermatophytosis, e.g., *Trichophyton indotineae* [17], *but*, *specifically*, *only as these diseases relate to neglected populations*. Table 1 details the expanded list, this being a revised version from our 2020 editorial [2]. As previously, the table includes columns with WHO/TDR and PLOS NTDs’ expanded lists.

**Table 1:** NTDs listed by the WHO and by *PLOS Neglected Tropical Diseases*.

Classes of NTDs	NTDs recognized by WHO	NTDs recognized by *PLOS Neglected Tropical Diseases*
Helminth infections (and their vectors)	• Dracunculiasis • Echinococcosis • Foodborne Trematodiases • Lymphatic filariasis • Onchocerciasis • Schistosomiasis • Soil-transmitted helminthiases • Taeniasis/Cysticercosis	All human helminth infections including but not restricted to: • Dracunculiasis • **Cystic, Alveolar, and Neotropical Echinococcosis**^**a**^ • Food-borne Trematodiases • Other food-borne helminthiases, including Trichinosis, Anisakiasis, Gnathostomiasis, etc. • Loaisis • Lympathic Filariasis • Onchocerciasis; **and other filarial infections**^**a**^ • Schistosomiasis • Soil-transmitted helminthiases (Ascariasis, Hookworm Disease, Trichuriasis, Strongyloidiasis) • Taeniasis-Cysticercosis • Toxocariasis and other *larva migrans*, e.g. Lagochilascariasis
Protozoan infections (and their vectors)	• Chagas disease • Human African trypanosomiasis • Leishmaniasis	• Amebiasis • Balantidiasis • Chagas Disease • Giardiasis • Human African Trypanosomiasis • Human babesiosis • Leishmaniasis • *Plasmodium vivax*, *P*. *knowlesi* and other non-*P*. *falciparum* malarias
Bacterial infections (and their vectors)	• Buruli ulcer • Leprosy • Noma • Trachoma • Yaws	• *Bartonella* • Bovine Tuberculosis in Humans • Buruli Ulcer • Cholera • Enteric pathogens (*Shigella*, *Salmonella*, *E*. *coli*) • Leprosy • Leptospirosis • Melioidosis • Noma • Relapsing Fevers caused by species of *Borrelia* • Rickettsial infections including Rocky Mountain spotted fever • Trachoma • Strep A *Streptococcus* • Q fever • Yaws and other tropical treponematoses–Bejel, Pinta
Fungal infections	• Mycetoma, chromoblastomycosis, and other deep mycoses	• Mycetoma, chromoblastomycosis, **talaromycosis, mucormycoses, sporotrichosis, *Trichophyton indotineae***, paracoccidiomycosis **and other emergent dermatophytoses and systemic mycoses**^**a**^
Viral infections (and their vectors)	• Dengue and Chikungunya • Rabies	• Arboviral infections including Dengue, Chikungunya, Zika, **Oropuche**^**a**^, Japanese encephalitis, Jungle yellow fever, West Nile Virus, and others • *Dabie bandavirus* (SFTS * **virus)**^**a**^ • Enterovirus 71 and related viruses • **Mpox**^**a**^ • Nipah and other *Henipavirus* spp. • Hantaviruses • HTLV-1, HTLV-2 and other non-HIV retrovirus infections • Rabies • Rift Valley fever • Other viral hemorrhagic fevers (VHFs) including Ebola and Marburg
Ectoparasitic infestations	• Scabies and other ectoparasitoses	• Scabies, Myiasis and other ectoparasitoses**
Non-infectious diseases or conditions	• Snakebite envenoming	• Podoconiosis • Snakebite envenoming • Scorpion envenoming

*PLOS Neglected Tropical Diseases* focuses on human disease, and although we are concerned with One Health aspects, infections that only afflict livestock and other animals are not in scope.

*severe fever with thrombocytopenia syndrome

**excluding head lice infestation

^a^newly added NTDs are noted in bold.

Under a new editorial structure, our editorial board now consists of small teams of Section Editors (SEs) specifically handling the above-mentioned major disease themes and working with an expanded cadre of Academic Editors (AEs). The AEs handle the manuscript consideration and review related process. By making these changes, we aspire to provide a more efficient service to our community and readers.

## Perspective

The scope of PLOS NTDs remains dynamic. Both we and WHO have expanded NTDs lists over the past few years (Table 1), and to reiterate our concluding comments from our 2020 editorial [2], PLOS NTDs is a community journal, and in that context, our aim is to be accessible and responsive to the NTD scientific community with respect to the journal’s scope. We have welcomed your feedback and been educated by you—the NTDs scientific community—on current topics and gaps in earlier NTDs lists. We continue to encourage submission of manuscripts with viewpoints and other front matter articles, including editorials or formal comments, making the case for or against a yet not-listed condition, and whether it qualifies as an NTD for inclusion in the journal’s scope. Our ultimate goal is to promote global and equitable health for all.
